# The Proportion of Female Physician Links With Advanced Educational Opportunity for Female and by Female

**DOI:** 10.15171/ijhpm.2019.147

**Published:** 2020-01-11

**Authors:** Yuki Senoo, Morihito Takita, Akihiko Ozaki, Masahiro Kami

**Affiliations:** ^1^Faculty of Medicine, Comenius University, Bratislava, Slovakia.; ^2^Medical Governance Research Institute, Tokyo, Japan.; ^3^Department of Breast Surgery, Jyoban Hospital of Tokiwa Foundation, Fukushima, Japan.

**Keywords:** Gender Gap, Advanced Education, Female Physician, Gender Inequality

## Abstract

**Background:** The overall proportion of female physician is increasing worldwide. However, its ratio exhibits a substantial diversity among each member country of Organisation for Economic Co-operation and Development (OECD). This study aimed to reveal the social factors of countries associated with the percentage of female physicians.

**Methods:** We retrieved the percentage of female physicians and social characteristic which may affect the ratio of female physicians of 36 OECD countries in 2016 or nearest year from the World Bank Open Data. Multivariate regression analysis was performed after univariate evaluations with Spearman’s coefficient to explore correlation of social variables with the proportion of female physicians.

**Results:** The percentages of female adolescents who dropped out of school before lower secondary school, female population that attained or completed Master’s or equivalent degree, female labour force, and female academic staff in tertiary education showed statistically significant correlation with proportion of female physicians (Spearman coefficient = -0.527, 0.585, 0.501, and 0.499; *P* =.01, .001, .002, and .008). Female’s educational attainment at least Master’s or equivalent and that of female academic staff at tertiary education were selected after multivariate analysis.

**Conclusion:** Our study revealed the relationships between advanced education opportunity and female participation in academic positions with the percentage of female physicians. Our research is limited in the difficulty to evaluate physicians’ working hours in spite of its possible effect. Further studies with qualitative assessment are warranted to explore the detail reasons to cause gender gap in physician.

Over the last several decades, social participation of women has been progressing worldwide. In fact, female to male ratio of labour force participation rate in high income countries has increased to 0.77 in 2018 from 0.67 in 1990, reported by the International Labour Organization.^1^ Similar trends of increased participation of female for medical workforce have been observed. In the United States, for instance, the proportion of licensed female physician was 35% in 2018, which increased by 37% from 2010.^2^ The female population of medical-school graduates has also increased as 46% in 2015 while 36% in 1990.^3^

The proportion of female physician, however, is diverse by country, ranging from 21% in Japan to 74% in Latvia among the Organisation for Economic Co-operation and Development (OECD) countries (Figure).^4^ This fact suggests that women’s status in the healthcare workforce could be impacted by cultural environments and women’s representation in individual countries. Thus, we determined whether the country characteristics could be correlated with the proportion of female physicians in this study.

**Figure:**
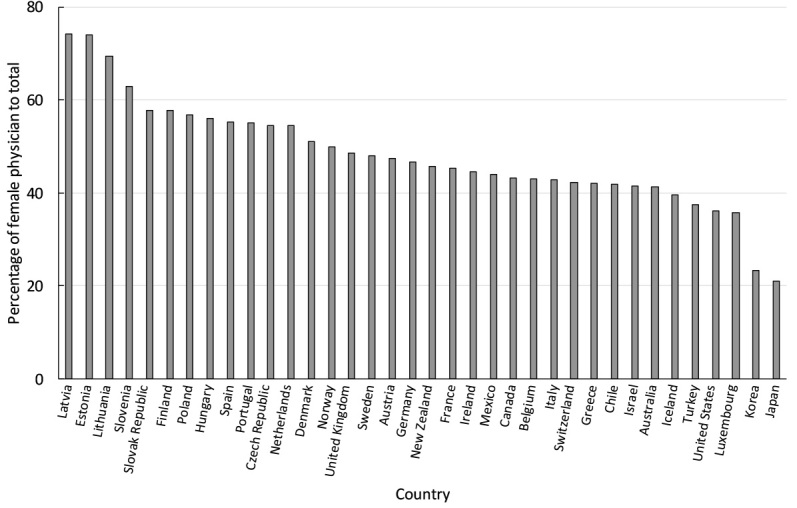
Proportion of Female Physicians.
The percentage of female physicians to total is presented by country in accordance with *OECD Health Statistics 2019* updated on November 15, 2019. The most recent data was implemented in the figure.

All 36 OECD countries were analysed in this study. All statistical analyses were made by IBM SPSS 25 (IBM, Armonk, NY) and statistical significance was considered when *P*  < .05. We tested a total of 146 variables from the World Bank Open Data for gender equality with Spearman correlation coefficients as univariate analysis (Supplementary file 1).^5^ After careful determination for duplicity or relationship between variables tested, the following variables were selected for multivariate evaluation; percentages of female adolescents out of secondary school, female children out of primary school, female’s educational attainment at least Master’s or equivalent, female labour force, and female academic staff at tertiary education. The multivariate linear regression analysis with stepwise selection was performed after we confirmed that the proportion of female physicians by country could follow a normal distribution determined by Shapiro–Wilk test. The regression model chose the percentage of female’s educational attainment at least Master’s or equivalent and that of female academic staff at tertiary education for significant contribution factors of female physician ratio (standardized beta coefficient = 0.44 and 0.48, *P*  = .02 and 0.01, respectively; Adjusted *R* square = 0.51).

Our study revealed that the female physician ratio could link with advanced educational opportunity for female and the proportion of female academic staff in advanced educational institute although further investigation with larger number of countries and longitudinal evaluation are necessary. Our results suggest the importance of enhancing advanced educational opportunity for women and education given by women to enable higher proportion of female physicians. More participation of women in advanced education may make better quality of healthcare service since it was reported that female physician performed their practice with smaller medical payments and fewer clinic visits compared to the male physicians.^6^

We focused on common social characteristics which may associate with the proportion of female physicians across OECD countries. In this context, we also evaluated if a comprehensive indicator for gender barriers known as the Global Gender Gap Index could be correlated with female physician ratio,^7^ while no significant correlation was seen (*r*  = 0.259 and *P*  = .15). The potential gender barriers in individual country, for instance, religions, regulations and policies, might influence female physician ratio. Japan and Korea are the lowest two countries (21% and 22% of female physicians to total, respectively), and are both in East Asia, which have distinct cultural and religious aspects to European countries included in our study. The gender discrimination in the admission examination of medical schools has been reported in Japan.^8^ Moreover, a recent study suggested that mistreatment, including sexual harassment, verbal and physical abuse based on gender, is more likely to cause mental exhaustion and burnout among female residents than male.^9^ While it is difficult to cross-sectionally evaluate and compare the degree of mental exhaustion based on gender discrimination, this variation may develop to a diverse gender gap observed among physicians in OECD countries. Further studies with qualitative assessment are warranted to explore the detail reasons to cause gender gap in physician.

## Acknowledgements

The authors thank Dr. Shuhei Nomura for his constructive opinions and insights.

## Ethical issues

Not applicable.

## Competing interests

AO reports personal fees from MNES Inc., outside of the submitted work. All other authors declare that they have no competing interests.

## Authors’ contributions

YS, MT, AO, and KM wrote the manuscript. All authors contributed to conception and design of the study, and critical revision of the paper. All authors read and approved the final manuscript.

## Authors’ affiliations

^1^Faculty of Medicine, Comenius University, Bratislava, Slovakia. ^2^Medical Governance Research Institute, Tokyo, Japan. ^3^Department of Breast Surgery, Jyoban Hospital of Tokiwa Foundation, Fukushima, Japan.

## Supplementary files


Supplementary file 1 contains Table S1.
