# Supplementary material for: The Proportion of Female Physician Links With Advanced Educational Opportunity for Female and by Female
Source: Int J Health Policy Manag. 2020 Jan 11;9(9):411–2. doi: 10.15171/ijhpm.2019.147 (PMC7557424; doi:10.15171/ijhpm.2019.147)
Supplement: Supplementary file 1 — contains Table S1. [file ijhpm-9-411-s001.pdf]

Supplementary file 1

Table S1. Spearman Correlation Coefficients for Proportion of Female Physicians

Variables	Spearman's correlation coefficient	p value
Account ownership at a financial institution or with a mobile-money-service provider (% of population ages 15+)	-0.022	0.901
Account ownership at a financial institution or with a mobile-money-service provider, female (% of population ages 15+)	-0.011	0.948
Account ownership at a financial institution or with a mobile-money-service provider, male (% of population ages 15+)	-0.036	0.835
Account ownership at a financial institution or with a mobile-money-service provider, older adults (% of population ages 25+)	-0.084	0.632
Account ownership at a financial institution or with a mobile-money-service provider, poorest 40% (% of population ages 15+)	0.002	0.990
Account ownership at a financial institution or with a mobile-money-service provider, primary education or less (% of population ages 15+)	-0.045	0.797
Account ownership at a financial institution or with a mobile-money-service provider, richest 60% (% of population ages 15+)	-0.001	0.994
Account ownership at a financial institution or with a mobile-money-service provider, secondary education or more (% of population ages 15+)	0.004	0.983
Account ownership at a financial institution or with a mobile-money-service provider, young adults (% of population ages 15-24)	-0.019	0.914
Adjusted net enrollment rate, primary (% of primary school age children)	0.121	0.508
Adjusted net enrollment rate, primary, female (% of primary school age children)	0.148	0.434
Adjusted net enrollment rate, primary, male (% of primary school age children)	0.120	0.528
Adolescents out of school (% of lower secondary school age)	-0.164	0.362
Adolescents out of school, female (% of female lower secondary school age)	-0.527	0.010
Adolescents out of school, male (% of male lower secondary school age)	-0.467	0.025

Age dependency ratio (% of working-age population)	0.075	0.664
Age dependency ratio, old (% of working-age population)	0.385	0.021
Age dependency ratio, young (% of working-age population)	-0.241	0.157
Births attended by skilled health staff (% of total)	-0.048	0.847
Cause of death, by communicable diseases and maternal, prenatal and nutrition conditions (% of total)	-0.388	0.019
Cause of death, by injury (% of total)	-0.270	0.111
Cause of death, by non-communicable diseases (% of total)	0.546	0.001
Children out of school (% of primary school age)	-0.121	0.508
Children out of school, female (% of female primary school age)	-0.148	0.434
Children out of school, male (% of male primary school age)	-0.120	0.528
Children out of school, primary	-0.334	0.061
Children out of school, primary, female	-0.402	0.027
Children out of school, primary, male	-0.386	0.035
Contributing family workers, female (% of female employment) (modeled ILO estimate)	-0.259	0.127
Contributing family workers, male (% of male employment) (modeled ILO estimate)	-0.085	0.621
Contributing family workers, total (% of total employment) (modeled ILO estimate)	-0.166	0.334
Educational attainment, at least Bachelor's or equivalent, population 25+, female (%) (cumulative)	0.024	0.905
Educational attainment, at least Bachelor's or equivalent, population 25+, male (%) (cumulative)	-0.352	0.066
Educational attainment, at least Bachelor's or equivalent, population 25+, total (%) (cumulative)	-0.208	0.288
Educational attainment, at least completed lower secondary, population 25+, female (%) (cumulative)	0.301	0.112
Educational attainment, at least completed lower secondary, population 25+, male (%) (cumulative)	0.298	0.116

Educational attainment, at least completed lower secondary, population 25+, total (%) (cumulative)	0.289	0.128
Educational attainment, at least completed post-secondary, population 25+, female (%) (cumulative)	-0.038	0.840
Educational attainment, at least completed post-secondary, population 25+, male (%) (cumulative)	-0.255	0.174
Educational attainment, at least completed post-secondary, population 25+, total (%) (cumulative)	-0.145	0.445
Educational attainment, at least completed primary, population 25+ years, female (%) (cumulative)	0.417	0.038
Educational attainment, at least completed primary, population 25+ years, male (%) (cumulative)	0.432	0.031
Educational attainment, at least completed primary, population 25+ years, total (%) (cumulative)	0.404	0.045
Educational attainment, at least completed short-cycle tertiary, population 25+, female (%) (cumulative)	0.054	0.771
Educational attainment, at least completed short-cycle tertiary, population 25+, male (%) (cumulative)	-0.369	0.038
Educational attainment, at least completed short-cycle tertiary, population 25+, total (%) (cumulative)	-0.174	0.341
Educational attainment, at least completed upper secondary, population 25+, female (%) (cumulative)	0.417	0.020
Educational attainment, at least completed upper secondary, population 25+, male (%) (cumulative)	0.304	0.096
Educational attainment, at least completed upper secondary, population 25+, total (%) (cumulative)	0.379	0.036
Educational attainment, at least Master's or equivalent, population 25+, female (%) (cumulative)	0.585	0.001
Educational attainment, at least Master's or equivalent, population 25+, male (%) (cumulative)	0.315	0.096
Educational attainment, at least Master's or equivalent, population 25+, total (%) (cumulative)	0.490	0.007
Educational attainment, Doctoral or equivalent, population 25+, female (%) (cumulative)	-0.080	0.684
Educational attainment, Doctoral or equivalent, population 25+, male (%) (cumulative)	-0.314	0.103
Educational attainment, Doctoral or equivalent, population 25+, total (%) (cumulative)	-0.221	0.259
Employers, female (% of female employment) (modeled ILO estimate)	0.139	0.419
Employers, male (% of male employment) (modeled ILO estimate)	0.002	0.990

Employers, total (% of total employment) (modeled ILO estimate)	0.029	0.867
Labor force participation rate, female (% of female population ages 15+) (modeled ILO estimate)	0.064	0.711
Labor force participation rate, female (% of female population ages 15+) (national estimate)	0.038	0.828
Labor force participation rate, female (% of female population ages 15-64) (modeled ILO estimate)	0.312	0.064
Labor force participation rate, male (% of male population ages 15+) (modeled ILO estimate)	-0.335	0.046
Labor force participation rate, male (% of male population ages 15+) (national estimate)	-0.337	0.044
Labor force participation rate, male (% of male population ages 15-64) (modeled ILO estimate)	0.012	0.945
Labor force participation rate, total (% of total population ages 15+) (modeled ILO estimate)	-0.154	0.370
Labor force participation rate, total (% of total population ages 15+) (national estimate)	-0.126	0.464
Labor force participation rate, total (% of total population ages 15-64) (modeled ILO estimate)	0.215	0.208
Labor force with advanced education (% of total working-age population with advanced education)	0.100	0.579
Labor force with advanced education, female (% of female working-age population with advanced education)	0.346	0.049
Labor force with advanced education, male (% of male working-age population with advanced education)	-0.148	0.411
Labor force with basic education (% of total working-age population with basic education)	-0.359	0.043
Labor force with basic education, female (% of female working-age population with basic education)	-0.306	0.089
Labor force with basic education, male (% of male working-age population with basic education)	-0.382	0.031
Labor force with intermediate education (% of total working-age population with intermediate education)	0.323	0.067
Labor force with intermediate education, female (% of female working-age population with intermediate education)	0.310	0.079
Labor force with intermediate education, male (% of male working-age population with intermediate education)	0.145	0.420
Labor force, female (% of total labor force)	0.501	0.002
Labor force, total	-0.395	0.017

Law mandates equal remuneration for females and males for work of equal value (1=yes; 0=no)	0.161	0.348
Law mandates nondiscrimination based on gender in hiring (1=yes; 0=no)	0.119	0.489
Law mandates paid or unpaid maternity leave (1=yes; 0=no)	0.097	0.575
Legislation exists on domestic violence (1=yes; 0=no)	-0.268	0.113
Life expectancy at birth, female (years)	-0.372	0.026
Life expectancy at birth, male (years)	-0.489	0.002
Life expectancy at birth, total (years)	-0.508	0.002
Maternal mortality ratio (modeled estimate, per 100,000 live births)	-0.029	0.865
Mortality caused by road traffic injury (per 100,000 people)	-0.095	0.581
Mortality rate, adult, female (per 1,000 female adults)	0.356	0.068
Mortality rate, adult, male (per 1,000 male adults)	0.432	0.025
Mortality rate, infant (per 1,000 live births)	-0.070	0.683
Mortality rate, infant, female (per 1,000 live births)	-0.091	0.597
Mortality rate, infant, male (per 1,000 live births)	-0.072	0.678
Mortality rate, under-5 (per 1,000 live births)	-0.054	0.753
Mortality rate, under-5, female (per 1,000 live births)	-0.063	0.715
Mortality rate, under-5, male (per 1,000 live births)	-0.056	0.747
Nondiscrimination clause mentions gender in the constitution (1=yes; 0=no)	-0.344	0.191
Part time employment, female (% of total female employment)	-0.150	0.383
Part time employment, male (% of total male employment)	0.137	0.427
Part time employment, total (% of total employment)	-0.017	0.923

Progression to secondary school (%)	0.014	0.950
Progression to secondary school, female (%)	0.153	0.487
Progression to secondary school, male (%)	-0.058	0.793
Proportion of seats held by women in national parliaments (%)	0.194	0.256
School enrollment, preprimary (% gross)	-0.070	0.696
School enrollment, preprimary, female (% gross)	-0.167	0.354
School enrollment, preprimary, male (% gross)	-0.134	0.456
School enrollment, primary (% gross)	0.104	0.545
School enrollment, primary (% net)	0.149	0.409
School enrollment, primary (gross), gender parity index (GPI)	0.095	0.583
School enrollment, primary and secondary (gross), gender parity index (GPI)	0.081	0.637
School enrollment, primary, female (% gross)	0.118	0.492
School enrollment, primary, female (% net)	0.179	0.335
School enrollment, primary, male (% gross)	0.090	0.600
School enrollment, primary, male (% net)	0.160	0.390
School enrollment, primary, private (% of total primary)	-0.149	0.386
School enrollment, secondary (% gross)	0.332	0.048
School enrollment, secondary (% net)	0.066	0.706
School enrollment, secondary, female (% gross)	0.302	0.073
School enrollment, secondary, female (% net)	0.100	0.573
School enrollment, secondary, male (% gross)	0.365	0.028

School enrollment, secondary, male (% net)	0.083	0.641
School enrollment, secondary, private (% of total secondary)	-0.318	0.059
School enrollment, tertiary (% gross)	-0.165	0.344
School enrollment, tertiary (gross), gender parity index (GPI)	0.428	0.010
School enrollment, tertiary, female (% gross)	-0.052	0.768
School enrollment, tertiary, male (% gross)	-0.189	0.277
Self-employed, female (% of female employment) (modeled ILO estimate)	-0.160	0.350
Self-employed, male (% of male employment) (modeled ILO estimate)	-0.125	0.469
Self-employed, total (% of total employment) (modeled ILO estimate)	-0.134	0.435
Sex ratio at birth (male births per female births)	0.178	0.298
Share of youth not in education, employment or training, female (% of female youth population)	-0.092	0.597
Share of youth not in education, employment or training, male (% of male youth population)	-0.152	0.383
Share of youth not in education, employment or training, total (% of youth population)	-0.112	0.521
Suicide mortality rate (per 100,000 population)	0.274	0.106
Suicide mortality rate, female (per 100,000 female population)	-0.011	0.947
Suicide mortality rate, male (per 100,000 male population)	0.341	0.042
Tertiary education, academic staff (% female)	0.499	0.008
Unemployment, female (% of female labor force) (modeled ILO estimate)	0.237	0.165
Unemployment, female (% of female labor force) (national estimate)	0.233	0.172
Unemployment, male (% of male labor force) (modeled ILO estimate)	0.279	0.099
Unemployment, male (% of male labor force) (national estimate)	0.279	0.099

Unemployment, total (% of total labor force) (modeled ILO estimate)	0.253	0.137
Unemployment, total (% of total labor force) (national estimate)	0.253	0.137
Wage and salaried workers, female (% of female employment) (modeled ILO estimate)	0.160	0.350
Wage and salaried workers, male (% of male employment) (modeled ILO estimate)	0.125	0.469
Wage and salaried workers, total (% of total employment) (modeled ILO estimate)	0.134	0.435

Abbreviation: ILO, International Labour Organization.

The Spearman's coefficients for correlation with proportion of female physicians are listed. Variables are highlighted if the p value was less than 0.05.
